# Postpartum acute intrauterine inversion: A case report

**DOI:** 10.1097/MD.0000000000043013

**Published:** 2025-07-25

**Authors:** Geer An, Weihong Chen, Zhiru Liang, Liyan Zhang, Yanan Zhang, Haiyan Li, Jing Wang, Yanfei Wang

**Affiliations:** a Department of Science and Education, Xilingol League Central Hospital, Xilin Hot, Inner Mongolia, P.R. China; b Department of Clinical Pharmacy, Anxi County Hospital, Quanzhou, Fujian, P.R. China; c Department of Hepatobiliary Surgery, Xilingol League Central Hospital, Xilin Hot, Inner Mongolia, P.R. China; d Department of Obstetrics and Gynecology, Xilingol League Central Hospital, Xilin Hot, Inner Mongolia, P.R. China; e Department of Surgical Anesthesiology, Xilingol League Central Hospital, Xilin Hot, Inner Mongolia, P.R. China.

**Keywords:** clinical management, low-risk pregnancy, postpartum hemorrhage, third stage of labor, uterine inversion

## Abstract

**Rationale:**

Uterine inversion is a rare and dangerous obstetric emergency, often occurring in the third stage of labor and leading to severe postpartum hemorrhage. It is less common in low-risk pregnant women, yet its prompt management is crucial. This case report fills a gap in the literature by presenting a rare instance in a low-risk young female, offering clinical insights.

**Patient concerns:**

A low-risk young female had sudden uterine inversion during the third stage of vaginal delivery, with persistent bleeding from the placental site, causing distress and posing an immediate health threat.

**Diagnoses:**

Physical examination revealed an abnormal uterine position and inability to contract normally, leading to a diagnosis of severe postpartum hemorrhage due to uterine inversion.

**Interventions:**

Immediate medical intervention was carried out to reposition the inverted uterus and control bleeding. After the correction, appropriate measures were taken to ensure stability and prevent complications.

**Outcomes:**

The patient’s condition was successfully managed, with the inversion corrected and bleeding stopped. Follow-up showed significant improvement and no long-term complications.

**Lessons:**

This case emphasizes vigilance for rare complications even in low-risk pregnancies and the need for prompt, coordinated intervention. It contributes to knowledge on uterine inversion management for researchers and clinicians.

## 1. Introduction

Uterine inversion is a rare but severe obstetric complication characterized by the collapse of the uterine fundus into the uterine cavity; accompanied by partial or complete eversion of the uterus.^[1]^ Based on the severity of the inversion of the uterine fundus, it can be classified into 4 types: Type I, where the extension of the uterine fundus does not exceed the cervical os; Type II, where the uterine fundus inverts to the cervical os but does not reach the vaginal orifice; Type III, where the uterine fundus inverts to the vaginal orifice; and Type IV, where the uterine fundus extends beyond the vaginal orifice with concurrent reverse inversion of the vaginal wall. Uterine inversion during the puerperium is primarily observed during the third stage of labor. If not properly managed, it can lead to severe consequences, including maternal death.^[2–4]^ This case report provides a retrospective analysis of the clinical features, diagnosis, treatment methods, and prognosis of a patient with acute postpartum uterine inversion, offering a reference for researchers and clinicians in related fields.

## 2. Case presentation

The patient is a 28-year-old female who was admitted to the hospital on September 10, 2022, due to “38 + 6 weeks of amenorrhea, 1.5 hours of paroxysmal abdominal pain, and 1 hour of vaginal discharge.” The patient’s height is 163 cm, with a prepregnancy weight of 65 kg and a weight gain of 12 kg during pregnancy. The patient underwent regular prenatal examinations at our hospital, and her last menstrual period was consistent with the gestational week, confirming an expected delivery date of September 25, 2022. The patient conceived naturally and had no significant family history, surgical history, or known drug allergies. Upon admission, routine blood and urine tests, blood coagulation tests, biochemical tests, and other laboratory examinations showed no abnormalities. Obstetric ultrasound revealed a cephalic presentation, fetal heart rate of 141 beats per minute, biparietal diameter of 9.0 cm, femoral length of 6.9 cm, amniotic fluid index of 9.0 cm, and the placenta located on the posterior wall of the uterus. Diagnosis upon admission: 1. primigravida, 38 + 6 weeks of gestation, cephalic presentation in labor; 2. pregnancy complicated with positive Group B Streptococcus in the vagina.

The patient went into labor at 00:30 on September 17, 2022. By 02:00, the cervical os had dilated to 3 cm, and by 03:00, it was dilated to 8 cm. The cervical os was fully dilated at 05:00. At 06:34, a male infant weighing 3700 g was delivered vaginally with an episiotomy, the Apgar score was 9 to 10. Routine intramuscular injection of 10 U of oxytocin was administered after delivery. At 06:40, the midwife noticed dark red blood flowing from the vagina. The uterus fundus was massaged, and the placenta was delivered with controlled traction on the umbilical cord. The placenta was naturally delivered at 06:44, with an umbilical cord length of 55 cm. Upon examination, the placenta and membranes were intact. Furthermore, during the examination of the soft birth canal, the cervix was not found, and a solid spherical tissue, approximately 9 × 9 cm in size, with a hard texture and smooth surface, was palpable. The normal uterine morphology could not be felt in the lower abdomen. The patient did not complain of abdominal pain; however she had significant vaginal bleeding. Immediately, 2 intravenous access routes were established, continuous electrocardiographic monitoring was initiated, and blood routine tests, coagulation tests, and cross-matching for blood transfusion were performed. Given the suspicion of uterine inversion, manual reduction was attempted but failed. At this point, the patient had a distressed appearance, with a blood pressure of 95/55 mm Hg and a heart rate of 125 beats per minute. The estimated blood loss was approximately 1200 mL, as a result, a diagnosis of postpartum hemorrhage was made. Blood tests showed hemotoglobin (HGB) at 114 g/L and hematocrit (HCT) at 33.6%, indicating a concentration of blood cells. 4 U of leukodepleted red blood cells and 400 mL of plasma were ordered.

**Figure 1.**
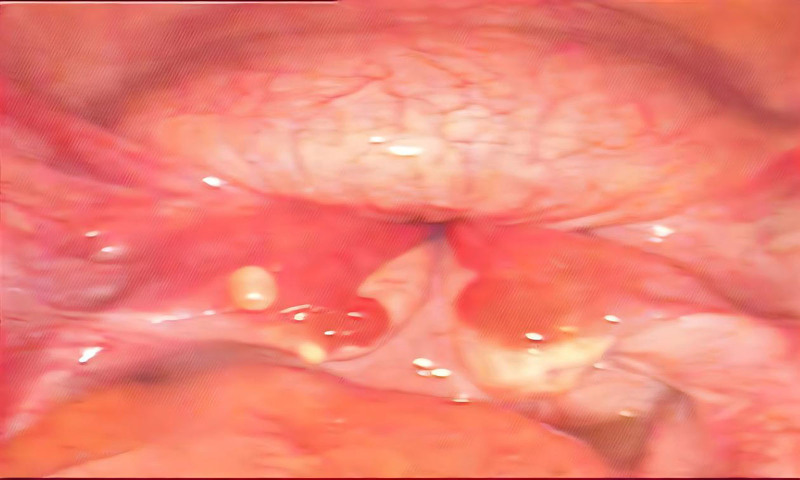
Imaging data of intraoperative neutron inversion.

At 07:20, the patient was transported to the operating room. presenting with intermittent active vaginal bleeding accompanied by the expulsion of blood clots. A third intravenous access route was immediately opened, and rapid fluid infusion with pressure was initiated. Her blood pressure dropped to 75/40 mm Hg, with a heart rate of 145 beats per minute and a respiratory rate of 32 breaths per minute. At 07:32, under general anesthesia, another attempt at vaginal manual reduction failed. An exploratory laparotomy for uterine reduction was performed at 08:02, revealing that both bilateral adnexa and the fundus of the uterus had completely inverted into the uterine cavity (Fig. 1). Manual reduction was successfully achieved immediately. At 08:06, 250 micrograms of carboprost tromethamine was administered intramuscularly into the uterine myometrium, and 100 µg of carbetocin was administered intravenously to promote uterine contraction. Since the blood products had not arrived, fluid infusion with pressure was continued. To reduce further bleeding, continuous compression and massaging of the uterus were performed, and the uterine body was wrapped in warm saline. At 08:10, leukodepleted red blood cells and plasma arrived, and 400 mL of fresh frozen plasma and 4 U of red blood cell suspension were administered. Additional blood cross-matching (4 U of red blood cells and 400 mL of plasma) was ordered, and uterotonic therapy was continued. Fifteen minutes after the previous use of Carboprost Tromethamine, another 250 µg was administered. Additionally, 20 U of oxytocin were injected intramuscularly into the uterine myometrium, and 6 U of pituitrin diluted in 40 mL were injected at multiple points in the uterine myometrium to enhance uterotonic therapy. Uterine contraction improved compared to before. At 08:45, 2.0 g of fibrinogen were infused. Simultaneously, absorbable sutures were used for compressive ligation of the anterior wall of the uterus, and the descending branches of the bilateral uterine arteries were ligated. At 09:00, another 250 µg of carboprost tromethamine were administered. In order to support the uterine myometrium and compress to stop bleeding, a water balloon was placed in the uterine cavity.

**Figure 2.**
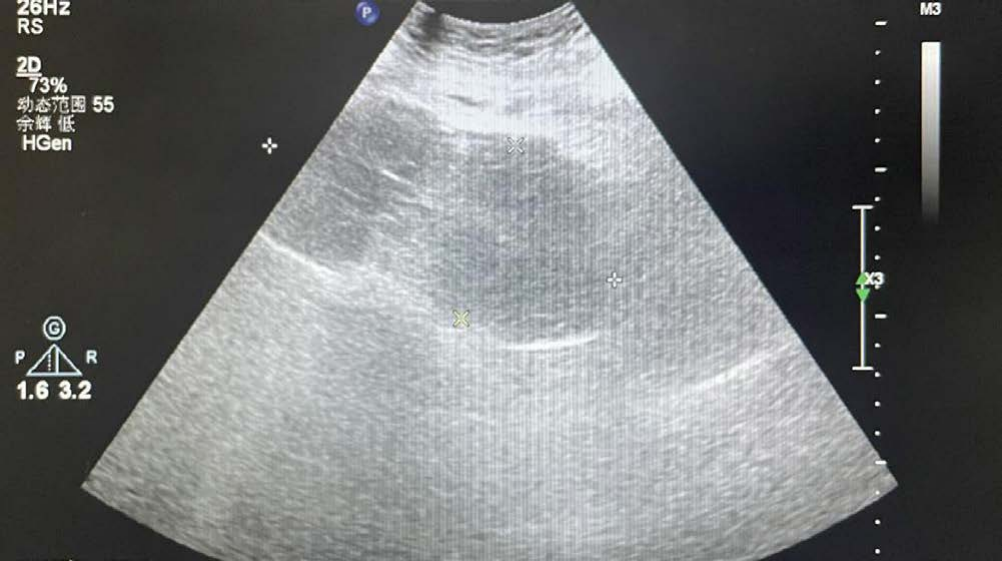
The patient’s uterine recovery 5 days after delivery.

**Figure 3.**
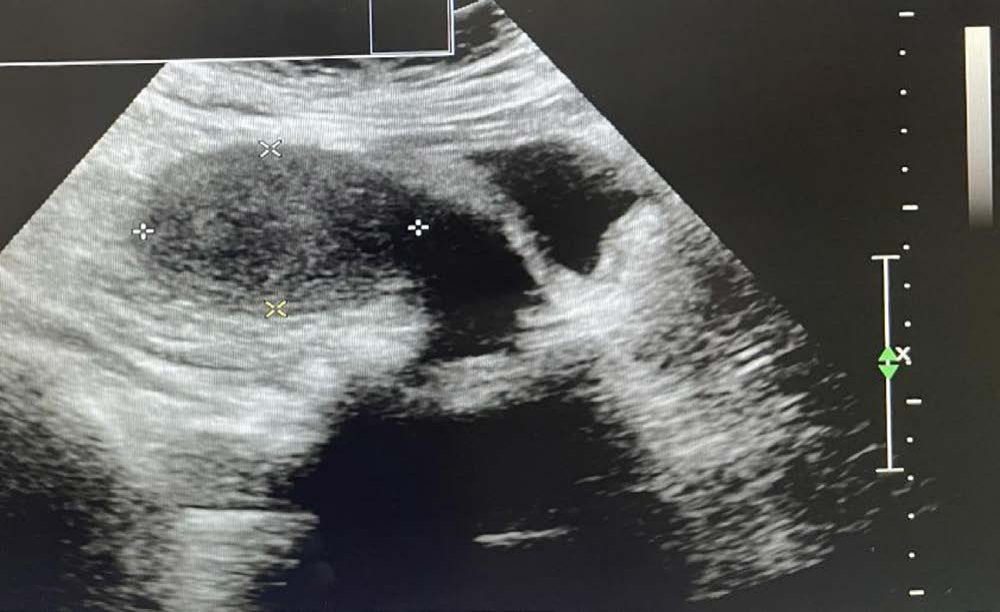
The patient’s uterine recovery 60 days after delivery.

After checking, we confirmed that there was no extension of the lateral episiotomy incision and no laceration of the birth canal. The assistant successfully placed a water balloon filled with 300 mL of liquid inside the uterine cavity inside the uterine cavity. To prevent the water balloon from falling out, an oval forceps was used to clamp the cervix at the 3 o’clock and 9 o’clock positions, thereby narrowing the cervical os. The oval forceps were removed at 10:30. Three medium-sized iodophor gauze pads were used to pack the vaginal fornix. After the above treatment, uterine contractions improved significantly, and bleeding was markedly reduced. The patient was then transferred to undergo re-disinfection and lateral episiotomy suturing. During this time, there was active bleeding from the needle holes, and considering the possibility of coagulation dysfunction due to significant bleeding, 400 mL of plasma was reinfused at 10:15. A small gauze was used to compress the perineal orifice for 10 minutes, and no active bleeding was observed. The surgery was completed. In total, there was 3000 mL of bleeding during labor, postpartum, and intraoperatively. The patient received 2000 mL of crystalloid, 1000 mL of colloid, 8 U of red blood cells, 800 mL of plasma, 2 g of fibrinogen, with a urine output of 180 mL. Blood pressure was 80/50 mm Hg, pulse rate was 85 beats per minute, and pulse oxygen saturation was 98%. A repeat blood test showed HGB at 73 g/L and HCT at 24.5%. After the surgery, the patient was transferred to the intensive care unit and received additional 8 U of suspended red blood cells, 400 mL of plasma, fluid infusion, monitoring of vital signs, various laboratory tests, uterotonic therapy, anti-inflammatory treatment, and other symptomatic treatments. On the first day after surgery, a repeat blood test showed HGB at 65 g/L and HCT at 19.0%, leading to the administration of an additional 4 U of suspended red blood cells. On the second day after surgery, the blood test showed HGB at 90 g/L and HCT at 26.0%, and the patient was transferred to a general ward to continue anti-inflammatory treatment for 5 days. A repeat abdominal ultrasound and uterine and bilateral adnexa ultrasound showed no obvious abnormalities. The patient was discharged on the seventh day after surgery in a stable condition. The uterine recovery at 5 days and 60 days postpartum is shown in Figs. 2 and 3, respectively.

## 3. Discussion

Uterine inversion is an extremely rare but potentially life-threatening obstetric complication, primarily occurring during the puerperium and less frequently outside of this period. The youngest reported case of uterine inversion was in an 11-year-old patient.^[5]^ Puerperal uterine inversion can be categorized into acute (occurring within 24 hours), subacute (occurring between 24 hours and 1 month), and chronic (lasting longer than 1 month) forms. Acute uterine inversion most commonly occurs during the third stage of labor. High-risk factors for uterine inversion include maternal connective tissue disorders, preeclampsia, anatomical abnormalities of the uterus, and the presence of uterine tumors. Additional factors are a weakened uterine wall at the placental site, placental location at the uterine fundus, a short umbilical cord, placental adhesion or implantation, excessive traction on the umbilical cord or abdominal pressure during the third stage of labor, cervical relaxation, macrosomia, and multiple gestations (such as twins).^[6,7]^ In this case, the patient was a young woman whose prenatal checkups had indicated low risk based on factors such as normal blood pressure, blood sugar levels, and fetal development. During the third stage of labor, the midwife pressed on the uterine fundus and pulled the umbilical cord before the placenta had completely detached, which may have been the primary cause of uterine inversion.

The incidence of puerperal uterine inversion is approximately 0.5 to 2.9 cases per 10,000 deliveries.^[8]^ The postpartum inversion of the uterus presents a series of characteristic clinical manifestations. For instance, during a lower abdominal palpation, the normal morphology of the uterine body cannot be felt, while an abnormal mass can be touched at the vaginal orifice, accompanied by irregular vaginal bleeding. In severe cases, it may even lead to hypovolemic shock. Bimanual examination is crucial in diagnosing uterine inversion and its severity, as it can accurately determine the status of uterine inversion. When diagnosis through physical examination alone is difficult, bedside ultrasonography should be immediately employed to rapidly confirm the diagnosis. During the management of this case, the midwife failed to detect the cervical structure when examining the soft birth canal but could touch a solid, spherical tissue. The normal shape of the uterus could not be felt in the lower abdomen, and there was irregular vaginal bleeding. These were typical clinical manifestations of uterine inversion, leading to an accurate diagnosis and subsequent prompt treatment. Given the specificity and severity of this condition, it is extremely crucial and urgent to enhance clinicians’ in-depth understanding of uterine inversion. Only by doing so can the condition be promptly detected and properly managed in clinical practice, thereby effectively avoiding adverse outcomes and ensuring the health and safety of patients.^[9,10]^

The successful treatment of this case reminds us that for patients with acute uterine inversion, healthcare providers are required to recognize the signs of uterine inversion early in clinical practice. For example, after the third stage of labor, attention should be paid to whether the patient experiences irregular vaginal bleeding, severe abdominal pain, and early signs of shock (such as pallor, hypotension, tachycardia, etc). Once uterine inversion with shock is confirmed, intravenous access should be immediately established, and crystalloid and colloid solutions should be rapidly infused. Blood transfusion therapy should be promptly initiated if necessary. While treating the shock, intravenous infusion of oxytocin should be immediately stopped, analgesics should be administered as soon as possible to relieve uterine spasms, and vaginal replacement should be promptly performed. If manual reduction fails, abdominal surgery for reduction should be promptly performed, as failure to do so may lead to severe postpartum hemorrhage and even hemorrhagic shock in postpartum women.^[11,12]^ In this case, we attempted nonsurgical methods to reduce the inverted uterus but were unsuccessful. Therefore, we adopted a scheme of manual abdominal reduction of the uterus under general anesthesia. After successful reduction, to effectively avoid the risk of secondary uterine inversion, a disposable balloon was used to compress the uterine cavity. At the same time, to prevent the water balloon from falling off, an oval forceps was used to clamp the cervix at the 3 o’clock and 9 o’clock positions, thereby narrowing the cervical os. This procedure not only provides effective support for the uterine myometrium, achieves hemostasis by compression, but also effectively prevents the recurrence of uterine inversion.^[13]^.

## 4. Conclusion

Through analyzing and discussing this case of acute postpartum inversion of uterus, we have a deep understanding of the specificity and severity of this disease. In clinical practice, high-risk factors for uterine inversion should be taken seriously, and the third stage of labor should be managed with caution. For example, irregular vaginal bleeding, abdominal pain, hypovolemic shock, and the presence of vaginal masses (such as those with specific size or shape) after delivery should prompt a high degree of vigilance for the possibility of uterine inversion. Early manifestations of uterine inversion should be identified as soon as possible, and shock should be actively corrected. Experienced senior doctors should be notified immediately, and manual reduction should be attempted as quickly as possible. We hope that this case report will help clinicians better understand the clinical manifestations and reduction process of uterine inversion.

## Acknowledgments

The author would like to thank the members of the research group for their demanding work.

## Author contributions

**Conceptualization:** Weihong Chen, Geer An, Zhiru Liang, Yanfei Wang.

**Data curation:** Weihong Chen, Geer An, Zhiru Liang, Yanfei Wang, Jing Wang.

**Formal analysis:** Weihong Chen, Geer An, Zhiru Liang, Yanfei Wang, Jing Wang.

**Funding acquisition:** Weihong Chen, Geer An, Zhiru Liang, Liyan Zhang, Yanfei Wang, Haiyan Li, Jing Wang.

**Investigation:** Weihong Chen, Geer An, Zhiru Liang, Liyan Zhang, Yanfei Wang, Yanan Zhang, Haiyan Li, Jing Wang.

**Methodology:** Weihong Chen, Geer An, Zhiru Liang, Liyan Zhang, Yanfei Wang, Yanan Zhang, Haiyan Li, Jing Wang.

**Project administration:** Weihong Chen, Geer An, Zhiru Liang, Liyan Zhang, Yanfei Wang, Yanan Zhang, Haiyan Li, Jing Wang.

**Resources:** Weihong Chen, Geer An, Zhiru Liang, Liyan Zhang, Yanfei Wang, Yanan Zhang, Haiyan Li, Jing Wang.

**Software:** Weihong Chen, Geer An, Zhiru Liang, Liyan Zhang, Yanan Zhang, Haiyan Li, Jing Wang.

**Supervision:** Weihong Chen, Geer An, Zhiru Liang, Liyan Zhang, Yanan Zhang, Haiyan Li, Jing Wang.

**Validation:** Weihong Chen, Geer An, Liyan Zhang, Yanan Zhang, Haiyan Li, Jing Wang.

**Visualization:** Weihong Chen, Geer An, Liyan Zhang, Yanan Zhang, Haiyan Li, Jing Wang.

**Writing – original draft:** Weihong Chen, Geer An, Yanan Zhang, Haiyan Li, Jing Wang.

**Writing – review & editing:** Weihong Chen, Geer An, Yanan Zhang, Haiyan Li, Jing Wang.
